# The Treatment of Locomotor Ataxy

**Published:** 1905-03-11

**Authors:** 


### THE TREATMENT OF LOCOMOTOR ATAXY.

It is hard to exaggerate the extent to which success in the treatment of grave, chronic organic disorders depends upon the mental attitude adopted by the practitioner in charge. Any patient who knows himself to be the victim of such a malady is sure to be oppressed, to some degree, with the sense of his unfortunate position, and the oppression is easily aggravated by pessimism in his medical attendant. It is easy for a doctor called upon to treat such an inveterate disease as locomotor ataxy to yield to a conviction of the hopelessness of cure, and to assume an attitude of what may be called vicarious stoicism. This, of course, is the worst thing possible for his patient, for it merely serves to indulge the melancholy outlook natural to a sick man. For this reason they cannot be sufficiently commended who set themselves to a determined attempt to achieve the maximum of alleviation possible under the circumstances, and the extent to which such alleviation may be carried is not seldom matter for gratified surprise. The mere fact that a skilled observer will undertake radical measures for his betterment is by itself sufficient, by pure suggestion, to lay the foundation in a patient for all the minor improvements in bodily health and spirits which, without pretending to be called cure, yet go far to make an afflicted existence tolerable and useful, if not actually a pleasure.

Dr. Hinsdale[1] has recently published what he calls a "hopeful case of locomotor ataxy," the details of which are as follows:—A man, aged 42, who denied that he had had syphilis, was none the less a typical example of locomotor ataxy. He suffered greatly from gastric crises and lightning pains, and was reduced to a miserable state of health. He was put to bed and underwent a "rest-cure," that is, rest, massage, and high feeding, with the addition of "sinusoidal" electricity, and the educational movements of the extremities which have for some time been practised in the treatment of this affection. In the matter of medication it was found that he was very intolerant of iodides, so he was treated with nitrate of silver and belladonna internally, and with antipyrin for the relief of the pains. In the course of three months he put on 38 lbs. of weight, the pains and ataxy vastly improved, and he was enabled to return to his usual occupation. Three years later he reappeared in a relapsed condition, his gastric crises being particularly severe. He was again submitted to similar treatment, with the addition of $\frac{1}{20}$th of a grain of mercuric chloride given under the skin twice daily. At the date of this treatment he was greatly bothered by inefficiency of his rectal sphincter, for the relief of which faradism was daily applied to the rectum. In nine weeks he gained 46 pounds in weight, and was sufficiently recovered in all respects to resume his occupation, although the sphincter trouble had not improved in proportion to the other symptoms.

Dr. J. Monroe Lieberman,[2] again, publishes some extraordinarily good results which he claims to have gained by exposing the region of the lumbar cord to the action of ultra-violet rays, results so good as to prompt scepticism. We do not propose to set out the details of the method, which may be found according to the above reference, but the routine, in the author's own words, is as follows:—(1) A warm half-bath at night before going to bed, with light massage; (2) ultra-violet rays in sittings of 10–30 minutes, thrice weekly; (3) static electricity by means of the Morton wave current or wooden brush, 15 to 20 minutes daily.

[1] Jour. of Amer. Med. Assoc., Feb. 18, 1905. [2] New York Med. Jour., Feb. 18, 1905.

